# Medical Convention

**Published:** 1846-03

**Authors:** 


MEDICAL CONVENTION.—A meeting of the Physicians of Rock River Valley was held, in pursuance of a call signed by sundry physicians of Rockford and vicinity, at the court house in Rockford, Illinois, on Tuesday, the 17th of February: Whereupon, on motion, Dr. G. HASKELL was called to the chair, and Dr. S. G. ARMOR appointed Secretary.

By request of the Chair, Dr. J. C. Goodhue stated the object of the meeting, which was the formation of an Association of the Physicians of Northern Illinois and Southern Wisconsin for mutual protection and improvement in the various branches of Medical, Surgical and Pharmaceutical knowledge —and presented, for the consideration of the Convention, a Constitution for the government of the Association, which was unanimously adopted.

The question, touching the *qualification* of members, and other matters of interest to the Society, then came up, and was discussed at considerable length, by Drs. Hulett, Haskell, Armor, Goodhue, Clark and others.

On motion, a committee, consisting of Drs. Hulett, Thomas, Clark, Catlin, and Manderville, were appointed by the chair to present to the Convention the names of officers for the Society for the ensuing year. The names of the following gentlemen were presented by the committee, and unanimously confirmed by the Convention :

J. C. GOODHUE, M. D., *President.*

J. HULETT, M. D., } *Vice*
G. HASKELL, M. D., } *Presidents.*

S. G. ARMOR, M. D., *Secretary and Treasurer.*

*Censors*—Lucius Clark, M. D.; A. M. Catlin, M. D.; Dr. A. Thomas.

On motion, Voted, That a committee of three be appointed by the chair to draft by-laws for the regulation and government of the Society, to be presented at the next annual meeting. Drs. Catlin, Clark and Goodhue, were appointed said committee.

On motion, Voted, That the President be requested to read a paper before the Society, at its next annual meeting, on some medical subject.

On motion, Voted, That the Secretary be requested to give a general call to physicians of Northern Illinois and Southern Wisconsin, to meet with us at our next annual meeting in May.

On motion, Voted, That the proceedings of this meeting be signed by the President and Secretary, and published in the Illinois Medical and Surgical Journal, and the papers in this portion of the State and Territory.

On motion, The meeting adjourned to meet in Rockford, Illinois, on the third Tuesday in May.

GEORGE HASKELL, *Pres't.*

SAMUEL G. ARMOR, *Sec'y.*

---

In obedience to a resolution of the "Rock River Medical Society," I am instructed to extend a general call to all physicians, of reputable standing in their profession, in Northern Illinois and Southern Wisconsin, to meet with us at the next annual meeting of the Society, as business of importance, relative to the adoption of a code of by-laws for the regulation and government of the Society, the election of officers, &c., will be acted on at that time. The object of the Association, as expressed in the Constitution adopted by the Society, is "for mutual improvement in the various branches of Medical, Surgical, and Pharmaceutical knowledge." The Society has been organized under favorable auspices, and promises to be of lasting benefit to the Profession in this part of the State and Territory. We ask to be protected by no *legal* enactments; neither do we require any tests of membership other than QUALIFICATION. The *evidence* of this will be, either the degree of DOCTOR OF MEDICINE, conferred by some respectable Medical College; the presentation of a License or Dimploma from some similar Medical Association; or a satisfactory examination before the board of Censors. More than this the Society cannot reasonably require;—less, we think, would fail in carrying out the true objects of such associations.

We believe we have begun right in this matter. Every subject connected with the proper organization of the Society, was candidly and freely discussed, and every difficulty, so far as they could be ascertained in the past history of similar associations, was satisfactorily removed. We now ask the co-operation of our brethren of the Profession in furthering the objects of the Association. We extend the invitation to every true lover of Medical Science, and indulge the hope that we shall be well represented at our annual meeting, at this place, on the third Tuesday of May.

SAMUEL G. ARMOR,
*Secretary of the Society.*

